# Effects of *Alismatis Rhizoma* and *Rhizoma Smilacis Glabrae* Decoction on Hyperuricemia in Rats

**DOI:** 10.1155/2019/4541609

**Published:** 2019-08-15

**Authors:** Shupin Cheng, Hanxiao Sun, Xiaomeng Li, Jiuming Yan, Zihao Peng, Yiping You, Lishi Zhang, Jinyao Chen

**Affiliations:** Food Safety Monitoring and Risk Assessment Key Laboratory of Sichuan Province, Department of Nutrition, Food Safety and Toxicology, West China School of Public Health/West China Fourth Hospital and Healthy Food Evaluation Research Center, Sichuan University, Chengdu 610041, China

## Abstract

The combination of *Alismatis Rhizoma* (AR) and *Rhizoma Smilacis Glabrae* (RSG), as Chinese herb medicine, has been used for their uric acid-lowering effect. However, the effects and mechanism of the combination of the two medicines have not been fully reported. Therefore, to explore the effects of AR-RSG combination decoction on the treatment of chronic hyperuricemia (HUA) in rats as well as the underlying mechanisms, in this study, at the first stage, a long-term HUA rats model was established by gavage of oteracil potassium plus adenine; allopurinol was used as the positive control, and the uric acid-lowering effects of AR or RSG decoction alone with low and high dose were evaluated, respectively. Serum uric acid (UA) and xanthine oxidase (XOD) were determined mainly, and pathological analysis of the kidney and liver was carried out after sacrifice of the animals. And then, at the second stage, four dose groups of AR-RSG combination treatment were investigated in HUA rats. In addition to the indicators measured at the first stage, the expression of urate anion exchanger 1 (URAT1) in rat kidney was determined by immunohistochemistry. We discovered that the UA levels of the model group in both stages were significantly and steadily higher than those of control groups. AR and RSG alone or in combination possess ability to decrease serum UA level of HUA rats, with effects more marked in the combination groups. The uric acid-lowering mechanism of AR-RSG combination may be related to its inhibiting activity of XOD, improving kidney damage and downregulating the expression of URAT1 in kidney.

## 1. Introduction

Hyperuricemia (HUA) refers to an abnormal state in which the fasting serum uric acid value is higher (7.0 mg/dL) under normal diet [1–3]. HUA is the biochemical basis of gout and a risk factor of other chronic diseases as well (e.g., acute and chronic kidney diseases, diabetes mellitus, coronary heart diseases, and metabolic syndrome) [4–8]. Currently, the medications used for lowering uric acid are mainly western pharmaceuticals, which if used in long term may cause adverse outcomes. For example, allopurinol has been reported to induce hypersensitivity reaction [9], benzbromarone may cause cells apoptosis and has certain hepatotoxicity [10], and uricase may lead to severe immunogenic and hemolytic reactions [11]. On the contrary, traditional Chinese medicine (TCM) has been used to treat gout for almost a thousand years and shows promising curative effect, and some of them are even as effective as western medicines [12]. A recent meta-analysis of 11 randomized controlled clinical trials showed that the efficacy of TCM is similar to that of western pharmaceuticals in treating HUA [12]. TCM, with natural sources and mild effects, may be more promising on long-term maintenance of serum uric acid (UA) level. Lu et al. has analyzed the role of TCM used for HUA and gout and found that *Alismatis Rhizoma* (AR) and *Rhizoma Smilacis Glabrae* (RSG) were frequently used in the treatment of HUA and gout [13].

However, due to the unclear material basis and mechanism of action of TCM, there is a certain gap in the application between TCM and western pharmaceuticals such as allopurinol currently used in clinical practice. In addition, problems such as nonuniform criteria of diagnosis and efficacy evaluation, lack of mechanism studies, and unclear long-term efficacy studies also exist in the medicine development for HUA.

Animal models play an important role in the screening of drugs for HUA and the preliminary exploration of the mechanism of action. At present, there are a number of methods to establish animal models of HUA. Rats and mice are used as model animals commonly, and the common model drugs are adenine, oteracil potassium, and so on. Studies have shown that modeling with a single drug often fails to achieve good results; currently, the combination of modeling agents has been mostly adopted, which could maintain longer and higher serum UA [14]. It should be noted that most animal models established before were not suitable for screening slow-acting agents such as TCM because of short-time treatment for both modeling and intervention [15–17]. Huo et al. from our research group have published the prophase study on establishment of rat models for screening slow-acting drugs of hyperuricemia before [18]; our previous reports indicate that the combination of adenine and oteracil potassium can establish a stable rat model of chronic HUA and allopurinol can be used as an effective positive control [18]. Therefore, based on the rat model we have established already, the uric acid-lowering effects of AR and RSG decoction alone were investigated firstly in this study and then based on the results of the first stage, the combination of AR-RSG decoction was studied in order to provide a basis for the subsequent research and the development of TCM prescriptions for UA lowering.

## 2. Materials and Methods

### 2.1. Reagents

Oteracil potassium (CAS no. 2207-75-2) was purchased from Jinan Chenghui Shuangda Chemical Co., Ltd. (Jinan, Shandong, China). Adenine (CAS No. 73-24-5) was purchased from Amresco L.L.C. (Solon, OH, USA). Allopurinol (CAS No. 315-30-0) was purchased from Tokyo Kakoki Co., Ltd. (Kamiina, Nagano, Japan). Adenosine deaminase (ADA) kit and xanthine oxidase (XOD) kit were purchased from Nanjing Jiancheng Bioengineering Research Institute (Nanjing, Jiangsu, China). Urate anion exchanger 1 (URAT1) primary antibody was purchased from Wuhan Sanying Biotechnology Co., Ltd. (Wuhan, Hubei, China). HRP-labeled goat anti-rabbit secondary antibody was purchased from Wuhan Saiweier Biotechnology Co., Ltd. (Wuhan, Hubei, China). All chemicals used were of analytical grade.

### 2.2. Plant Material

*Alismatis Rhizoma* (AR, zexie in Chinese) (batch number: 170208) and *Rhizoma Smilacis Glabrae* (RSG, tufuling in Chinese) (batch number: 170301) were purchased from Sichuan Guoqiang Chinese Herbal Pieces Factory (Chengdu, Sichuan, China) in March 2017.

### 2.3. Preparation of AR and RSG Decoction

The decoction administered to rats was prepared as comparable to real-life scenario. At the first stage, the proper amount of AR pieces was weighed, added with 10 times of water, soaked for 30 min, and decocted for 45 min in a gallipot. After collection of the liquid, an equal amount of water was added again and decocted for 45 min and then the two collections were combined together and concentrated to the required doses. RSG pieces were also prepared as stated above. At the second stage, AR and RSG pieces were weighed proportionally and mixed together and then the corresponding combination decoction concentrates were prepared in the same way.

### 2.4. Experimental Animals

Adult male SD rats (200 ± 20 g, SPF-grade) were procured from the Dashuo Laboratory Animal Reproduction Center (Chengdu, China) with animal license: SCXK (Chuan) 2013–24. Two batches of rats were used for two stages in this study. The rats were kept at the Animal Laboratory Center of West China School of Public Health (Chengdu, China) with license: SYXK (Chuan) 2013–011 and given free access to standard commercial rodent feed and drinking water. Animal treatment protocol followed the guidance of the Ethical Committee for Research on Laboratory Animals of Sichuan University and was in accordance with the international guidelines for laboratory animal use and care found in the European Community guidelines.

### 2.5. Treatment

The efficacy of AR or RSG alone was evaluated at the first stage. Rats were randomized to seven groups (*n* = 10), including control group (C), model group (M), positive control group-allopurinol group (A), low-dose AR group (AR-L), high-dose AR group (AR-H), low-dose RSG group (RSG-L), and high-dose RSG group (RSG-H). And then, the evaluation of efficacy of AR-RSG combinations was carried out at the second stage. Rats were randomly divided into seven groups (*n* = 10), namely, control group (C), model group (M), allopurinol group (A), and combination group 1 (PF-1), 2 (PF-2), 3 (PF-3), and 4 (PF-4).

All treatments were via oral gavage in a volume of 10 mL/kg·bw for 45 days. The modeling agent was dissolved with 1% CMC-Na. In the positive control group, allopurinol was dissolved with pure water. AR, RSG, and their combination treatment were the corresponding decoction concentrates. The details about the treatment are summarized in Table 1.

### 2.6. Samples Collection and Parameters Measurement

For two stages, body weight was measured and recorded before treatment on the first day of dosing and twice per week thereafter and prior to necropsy.

#### 2.6.1. Blood Collection and Measurement

Blood collection and indicators detection in two stages were the same. The rats were fasted eight hours prior to blood collection with free access to water. Peripheral blood from posterior venous plexus was collected on day 1 (as baseline), 15, 30, and 45 after animals were general anesthetized. After centrifugation at 1300 g for 10 min, the serum was isolated and stored at −80°C to determine the serum uric acid (UA), creatinine (Cr), and urea nitrogen (UN). In addition, on the 45^th^ day, serum liver biological indexes including alanine aminotransferase (ALT), aspartate aminotransferase (AST), and total bilirubin (T-BIL) and uric acid metabolism-related enzymes (XOD and ADA) were detected.

For treatment groups, the percentage of decrease in UA in these groups compared with corresponding model groups was calculated using the following equation on day 15, 30, and 45:

(1) $$\begin{array}{c} \text{percentage of decrease in UA }\left(\text{\%}\right)=\frac{\text{UA in model groups}-\text{UA in medicine groups}}{\text{UA in model groups}}\times 100\%. \end{array}$$

#### 2.6.2. Histopathology Analysis

After sacrificing, the livers and kidneys of the rats were excised and weighed. Livers and kidneys were fixed in 10% buffered formalin, embedded in paraffin, and stained with hematoxylin and eosin (H&E). Light microscopy studies were done under a photomicroscope (Olympus BX50F4, Japan). The extent of the tissue damage was evaluated by the Image-Pro Plus System 5.0 (Media Cybernetics Inc., Silver Spring, MD, USA). The images of whole tissue sections from all animals were captured under ×200 optical magnification. All histological determinations were done by a blinded observer.

#### 2.6.3. UART1 Expression in Kidney with Immunohistochemistry Analysis

Only in the second stage, immunohistochemistry was conducted to determine the expression of kidney urate anion transporter 1 (URAT1). Kidney tissues were dehydrated in a graded series of alcohols, embedded in paraffin, and sectioned at 3 *μ*m. After deparaffinization and rehydration, endogenous peroxidase activity was blocked by using 3% H_2_O_2_ solution for 25 min. Sections were then incubated for 30 min at room temperature in blocking solution containing 3% bovine serum albumin, followed by incubation with primary antibodies diluted in PBS at 4°C overnight (anti-URAT1 antibody, 1 : 50). Secondary antibody incubation was performed at room temperature for 50 min, followed by developing with DAB, restaining with hematoxylin, water washing, staining with blue dye, air drying, and film sealing. The tissues were observed under a microscope, and high-quality images were collected by CCD software (1600 × 1200 pixels); then, the integrated optical density (IOD) of the selected areas was measured by IPP program software.

### 2.7. Statistical Analyses

Results were expressed as mean values with standard deviations (mean ± SD). Statistical analysis was performed using Statistical Product and Service Solutions (SPSS) Version 20.0, and the level of significance was set at *α* = 0.05. Multiple means were compared using one-way analysis of variance (ANOVA) after checking for homogeneity of variance, or the Kruskal–Wallis rank sum test was performed. Comparison between groups was undertaken with Dunnett's test while *P* ≤ 0.05 in ANOVA.

## 3. Results

No rat died during the experiment both in the first and second stage. Later in the 45 consecutive days, yellow and clutter in furs, apathy, and burnout in spirit were observed in model groups, positive control groups, and all treatment groups except the control groups.

### 3.1. Results of the First Stage

#### 3.1.1. Body Weights and Organ Weights

At the first stage, body weights at sacrifice and relative liver/kidney-to-body weights of rats in each group are summarized in Table 2. Significant decrease (*P* < 0.05 or *P* < 0.01) of body weights was observed in rats of model and the treatment groups compared with those of control. Except for the AR-H group, the relative kidney weights of the other groups were significantly higher than those of control group (*P* < 0.05). Compared with those in group M, the body weight of rats in group RSG-H was significantly lower (*P* < 0.01) and the relative liver weight was significantly higher (*P* < 0.05).

#### 3.1.2. Necropsy and Histopathologic Findings

**Figure 1:**
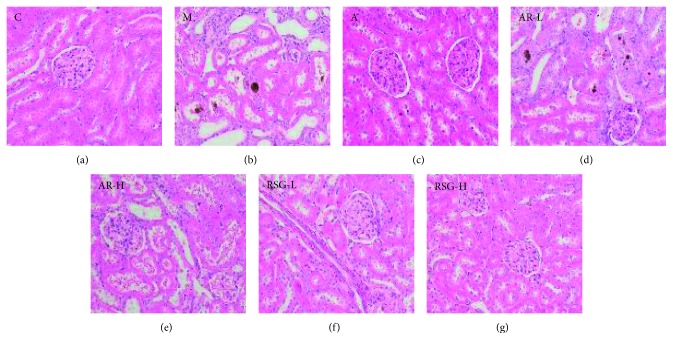
Hematoxylin and eosin stains of kidney sections of all groups in the first stage: (a) C, control; (b) M, model; (c) A, allopurinol; (d) AR-L, low dose of AR; (e) AR-H, high dose of AR; (f) RSG-L, low dose of RSG; (g) RSG-H, high dose of RSG (images under ×200 optical magnification).

At the first stage, the kidney tissue structure of rats in the control group was normal. In group M, the kidney volume was increased, with yellow vesicles or particles on the surface, moderate glomerular atrophy, moderate tubular dilation, epithelial necrosis, and exfoliation with severe inflammatory cell infiltration and more brown crystalline precipitation in the renal tubes. In the TCM groups (except group AR-L) and group A, there was mild inflammatory cell infiltration in the kidney tubules and only a small amount of crystallization was observed in the tubules, indicating their effect on alleviating kidney damage, as shown in Figure 1.

**Figure 2:**
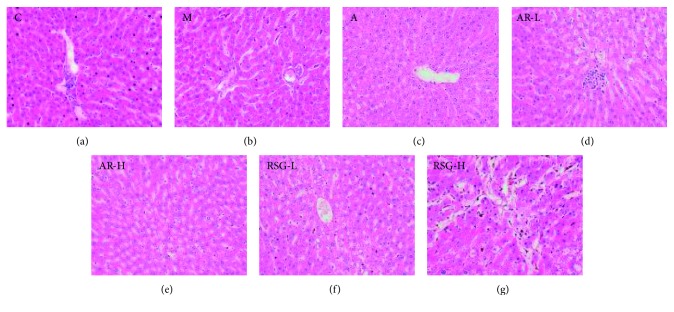
Hematoxylin and eosin stains of liver sections of all groups in the first stage: (a) C, control; (b) M, model; (c) A, allopurinol; (d) AR-L, low dose of AR; (e) AR-H, high dose of AR; (f) RSG-L, low dose of RSG; (g) RSG-H, high dose of RSG (images under ×200 optical magnification).

The pathological microscopic examination of the livers showed intercellular pigment crystallization in some areas with flaky necrosis in group RSG-H, as shown in Figure 2.

#### 3.1.3. Serum Liver Biological Indexes (ALT, AST, and T-BIL)

Levels of ALT, AST, and T-BIL in group RSG-H were significantly higher than those in group M, suggesting that there was liver damage of rats in the RSG-H group, as shown in Table 3.

#### 3.1.4. Serum UA, Cr, and Ur Levels

**Figure 3:**
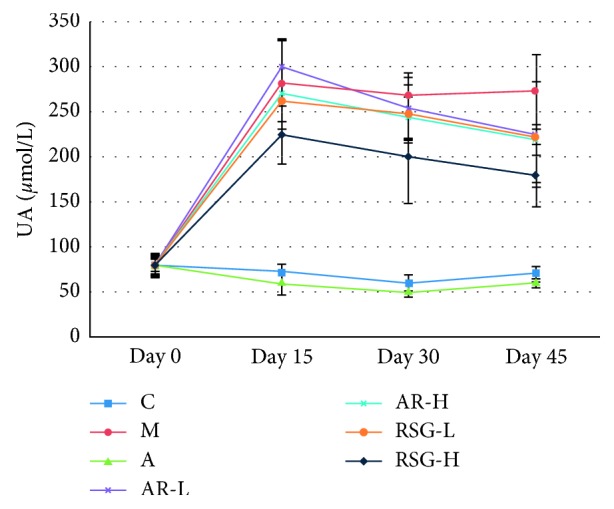
Serum UA levels in rats during 45 days in the first stage: C, control; M, model; A, allopurinol; AR-L, low dose of AR; AR-H, high dose of AR; RSG-L, low dose of RSG; RSG-H, high dose of RSG.

The level of UA of rats in group M was significantly higher than that in the control group, suggesting that the rats model of HUA was successfully developed and maintained till the 45^th^ day. The UA level in group A and RSG-H was significantly lower than that in group M on the 15^th^, 30^th^, and 45^th^ day (*P* < 0.05 or *P* < 0.01). The UA levels in groups AR-L, AR-H, and RSG-L were significantly lower than those in group M at the 45^th^ day (*P* < 0.05). The descent rate of UA in TCM groups was lower than that in group A, as shown in Figure 3. The percentage of decrease in UA is shown in Table 4.

The Cr and Ur levels in group M were significantly higher than those of the control group (*P* < 0.01). The Ur level in group A was significantly lower than that of group M (*P* < 0.01). In group RSG-H, the Cr levels, consistent with Ur levels, were all significantly lower than those in group M on the 30^th^ and 45^th^ day (*P* < 0.01), suggesting that RSG decoction may have protective effect on the kidney function of HUA rats, as shown in Table 5.

#### 3.1.5. Serum UA Metabolism-Related Enzyme (XOD and ADA) Levels

**Figure 4:**
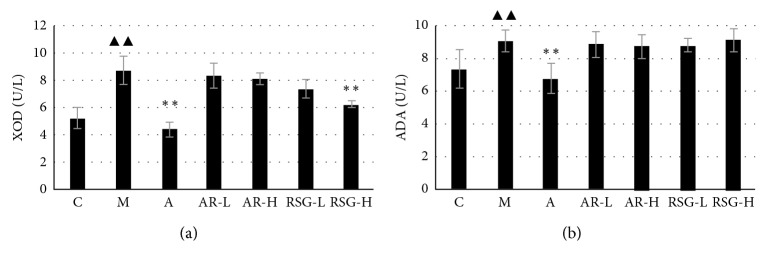
Serum XOD and ADA levels in rats with different treatment on the 45^th^ day in the first stage: C, control; M, model; A, allopurinol; AR-L, low dose of AR; AR-H, high dose of AR; RSG-L, low dose of RSG; RSG-H, high dose of RSG; XOD, xanthine oxidase; ADA, adenosine deaminase. ^▲▲^Significantly different from those of the control group at the level of *P* < 0.01. ^*∗∗*^Significantly different from those of the model group at the level of *P* < 0.01.

Serum XOD and ADA levels in group M were significantly higher than those in the control group. The levels of XOD and ADA in group A were significantly lower than those in group M. Only in group RSG-H, the XOD level was significantly lower than that in group M (*P* < 0.01), as shown in Figure 4.

### 3.2. Results of the Second Stage

#### 3.2.1. Body Weights and Organ Weights

Body weights at sacrifice and relative liver/kidney-to-body weights of rats in each group are summarized in Table 6. Compared with those of the control group, the relative kidney weights of the other groups increased significantly (*P* < 0.01) and there was no significant difference of body weights and relative liver weights among all groups (*P* > 0.05).

#### 3.2.2. Necropsy and Histopathologic Findings

**Figure 5:**
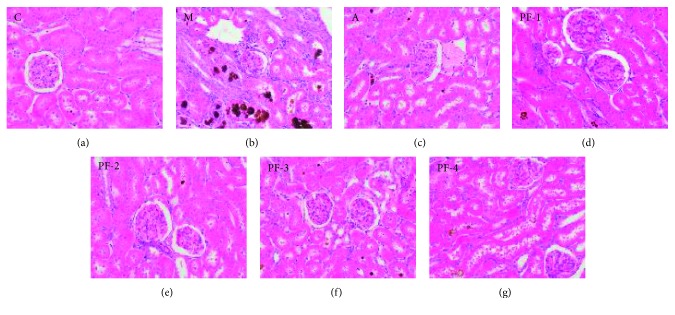
Hematoxylin and eosin stains of kidney sections of all groups in the second stage: (a) C, control; (b) M, model; (c) A, allopurinol; (d) PF-1, combination 1; (e) PF-2, combination 2; (f) PF-3, combination 3; (g) PF-4, combination 4 (images under ×200 optical magnification).

Kidney tissue structure of the control group was normal. In group M, the kidney volume was increased with yellow vesicles or gray-white particles on the surface, moderate atrophy of glomeruli, moderate dilatation of renal tubules, more necrosis and shedding of epithelium with severe inflammatory cell infiltration, and more brown crystalline precipitation in the tubes. Mild inflammatory cell infiltration was observed in kidney tubules of rats in PF-1, -2, -3, and -4 groups and group A, and only a small amount of crystallization was observed in the tubules, indicating their effects of alleviating kidney injury, but there was no significant difference among the treatment groups, as shown in Figure 5.

A small amount of inflammatory cell infiltration accompanied by mild cell atrophy and crystallization was observed in the liver of rats in group M, group A, and all TCM treatment groups (PF-1, -2, -3, and -4), without significant difference among these groups. The observation might be attributed to increased intake of adenine (picture not shown).

#### 3.2.3. Serum Liver Biological Indexes (ALT, AST, and T-BIL)

There was no statistical difference of serum ALT, AST, and T-BIL among all groups, as shown in Table 7.

#### 3.2.4. Serum UA, Cr, and Ur Levels

**Figure 6:**
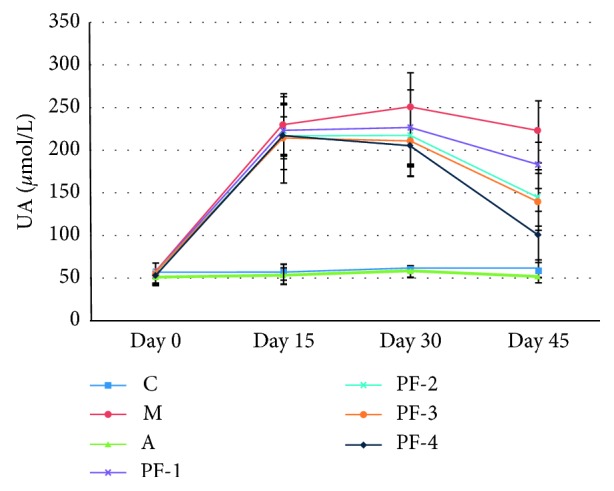
Serum UA levels in rats during 45 days in the second stage: C, control; M, model; A, allopurinol; PF-1, combination 1; PF-2, combination 2; PF-3, combination 3; PF-4, combination 4.

Levels of UA in group A was significantly lower than those in group M (*P* < 0.01) at any timepoint. The UA levels of group PF-4 on the 30^th^ and 45^th^ day were significantly lower than those in group M (for both, *P* < 0.05), while the UA levels of group PF-1, -2, and -3 were significantly lower than those of group M at the 45^th^ day (for all, *P* < 0.05). The UA level of group PF-4 decreased by more than 50% on the 45^th^ day, as shown in Figure 6. The percentage of decrease in UA is shown in Table 8.

The Cr and Ur levels in group M were significantly higher than those of the control group (*P* < 0.01), and the Cr and Ur levels in group A were significantly lower than those of group M (*P* < 0.01). The Cr levels of group PF-2, -3, and -4 were significantly lower than those of group M on the 30^th^ and 45^th^ day (*P* < 0.05 or *P* < 0.01). The Ur levels in all combination groups were significantly lower than those of group M on the 45^th^ day (*P* < 0.01), as shown in Table 9.

#### 3.2.5. Serum UA Metabolism-Related Enzyme (XOD and ADA) Levels

**Figure 7:**
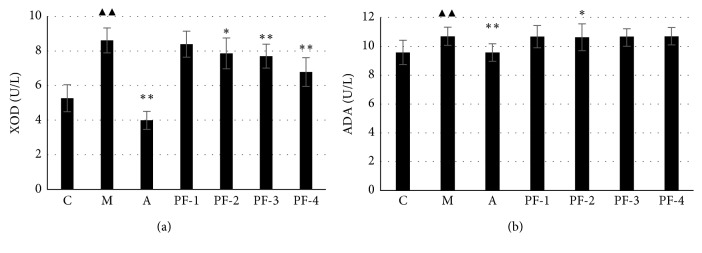
Serum XOD and ADA levels in rats with different treatment on the 45^th^ day in the second stage: C, control; M, model; A, allopurinol; PF-1, combination 1; PF-2, combination 2; PF-3, combination 3; PF-4, combination 4; XOD, xanthine oxidase; ADA, adenosine deaminase. ^▲▲^Significantly different from those of the control group at the level of *P* < 0.01. ^*∗*^ and ^*∗∗*^ indicate values significantly different from those of the model group at the levels of *P* < 0.05, *P* < 0.01, respectively.

Compared with those in the control group, the XOD and ADA levels in group M were significantly increased. The levels of XOD and ADA in group A were significantly lower than those of group M (*P* < 0.01). The XOD levels of group PF-2, -3, and -4 were significantly lower than those of group M (*P* < 0.05 or *P* < 0.01). The ADA level in group PF-2 was significantly lower than that in group M, as shown in Figure 7.

#### 3.2.6. Expression of URAT1 in Kidney

**Figure 8:**
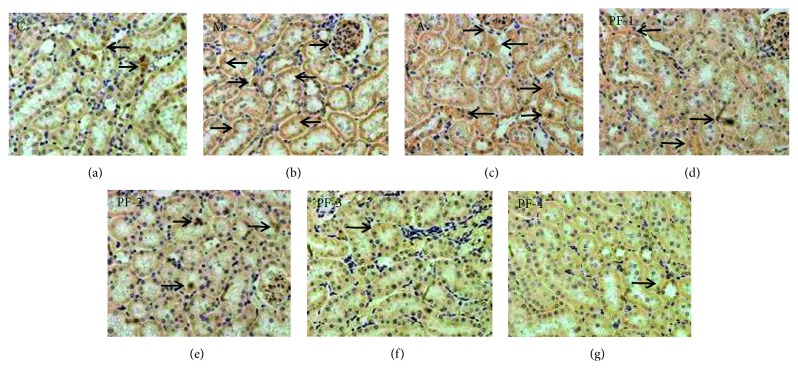
Immunohistochemical examination of URAT1 expression in kidney in the second stage: (a) C, control; (b) M, model; (c) A, allopurinol; (d) PF-1, combination 1; (e) PF-2, combination 2; (f) PF-3, combination 3; (g) PF-4, combination 4 (images under ×400 optical magnification).

**Figure 9:**
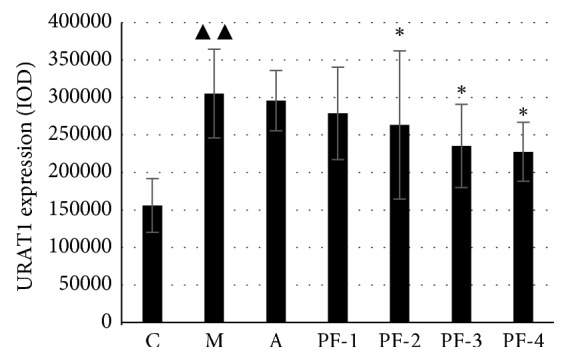
URAT1 expression in kidney (IOD) of rats on the 45^th^ day in the second stage: C control; M model; A allopurinol; PF-1, combination 1; PF-2, combination 2; PF-3, combination 3; PF-4, combination 4. ^▲▲^Significantly different from those of control group at the level of *P* < 0.01. ^*∗*^Significantly different from those of model group at the level of *P* < 0.05.

Expression of URAT1 was significantly increased in group M compared with the control group (*P* < 0.01). Compared with that of group M, the expression of URAT1 in group A and all TCM groups were decreased to different extent. Only in group PF-3 and PF-4, the expression of URAT1 was significantly decreased, as shown in Figures 8 and 9.

## 4. Discussion

Hyperuricemia is mainly determined by serum uric acid levels, the clinical symptoms of which are not obvious, but it is the biochemical basis and key risk factor of gout attack [19]. Some researchers have used TCM for treating gout and found that herbs such as *Alismatis Rhizoma* (AR), *Rhizoma Smilacis Glabrae* (RSG), and their extracts have certain effects on reducing UA [20–22]. As we all know, TCM treatment takes a longer time and shows slower progress than western medicine does, and the effect of TCM is less immediate but perhaps more substantial. Meanwhile, the modeling time for most HUA animal models at present is relatively short. Therefore, we established a long-term effective hyperuricemia rat model firstly [18] and then used the rat model to evaluate the efficacy of TCM in treating HUA. Considering that the way of taking TCM for most patients is decoction instead of single purification or several extracts, our study used the decoction of AR and RSG as interventional treatment, which has important practical significance to the research of TCM prescription of uric acid-lowering effects.

In this study, the UA levels of the model group in both stages were significantly and steadily higher than those of control groups at any timepoint, which were consistent with the results of model establishment we have published before [18], indicating that the model of HUA in rats was established successfully.

It is recognized that it would be better if the doses effective in animal experiments could be deduced to actual human intake. According to the relevant requirements of dose selection in the Chinese national standard of “functional evaluation procedures and testing methods for health food (2003),” in general, 5 to 20 times of the minimum recommended dose for human are suggested as the effective dose range for test substances of function evaluation in animal studies. Therefore, in the first stage, the low- and high-dose settings of AR or RSG were equivalent to 5 and 20 times of the minimum recommended dose for human (AR: 0.1 g/kg·bw; RSG: 0.25 g/kg·bw). The results showed that AR and RSG could reduce UA but were not as good as that of allopurinol. Serum Cr and Ur levels could reflect kidney damage, and serum AST, ALT, and T-BIL values could reflect liver damage. Compared with those of group M, high dose of RSG could significantly reduce serum Cr and Ur levels in HUA rats, but serum AST, ALT, and T-BIL levels in this group were significantly increased; combined with this, microscopic pathological examination showed liver damage, which suggests high dose of RSG could alleviate the degree of kidney damage in rats but might cause certain liver damage. XOD and ADA are both important enzymes involved in UA synthesis. Only high dose of RSG could significantly inhibit the content of XOD. RSG decoction may reduce serum UA by inhibiting XOD activity.

Results of the first stage showed that 0.50∼2.00 g/kg·bw of AR decoction and 1.25∼5.00 g/kg·bw of RSG decoction could reduce UA but the high dose of RSG caused liver damage. Therefore, combined with UA-lowering effect and its possible mechanism, in the second stage, 1.00 g/kg·bw of AR was used as the reference and the dose rate of AR: RSG = 1 : 1, 2 : 1, 1 : 2, and 2 : 2 was used for dose design.

Results showed that the four AR-RSG combination groups all had the effect of decreasing UA and, according to the declining trend of UA levels, it was speculated that the effect of reducing UA was time dependent. When the content of AR was the same (2.00 g/kg·bw), the drop of UA in group PF-2 and group PF-4 was greater than that in group AR-H (shown in Tables 4 and 8.), indicating that the effect of reducing UA of the combination group was better than that of the single drug. The four formulations can reduce the serum Cr and Ur levels to different degrees, and improve the kidney injury in rats. In the second stage, the XOD levels of groups PF-2, -3, and -4 were significantly lower than those in group M, suggesting that the UA-lowering effect of the combination may be related to decreasing XOD activity. The kidney damage of rats with HUA was all alleviated in combination groups, and the protective effect on the kidney may also be one of the mechanisms of the combination treatment to reduce UA.

Furthermore, URAT1 is a membrane transporter encoded by *SLC22A12* gene of the organic anion-encoding family *SLC22A*, which is mainly located in the lumen side of the brush border epithelial cells of the proximal convoluted tubule of the kidney cortex and involved in the resorption of uric acid, thereby regulating the concentration of serum uric acid [23]. Studies have shown that the loss of URAT1 function is strongly associated with kidney hypouricemia and the UA excretion rate of these people can even reach 100% [24, 25]. Inhibiting the expression of URAT1 can effectively reduce the serum uric acid level *in vivo*, which is an important approach to treat hyperuricemia. Studies have reported that TCMs such as *Rhizoma Smilacis Glabrae* and *Semen Plantaginis* can reduce the expression of URAT1 in HUA rats [26]. In our study, immunohistochemical results of kidney URAT1 in rats showed that PF-3 and PF-4 could significantly downregulate the expression of URAT1 in the kidney of HUA rats, suggesting that combination of AR and RSG could promote UA excretion by inhibiting the expression of URAT1 in the kidney of HUA rats. And according to the dose setting of combination groups, it can be speculated that the effect of RSG on inhibiting the expression of URAT1 may be stronger than that of AR.

## 5. Conclusion

Combination of *Alismatis Rhizoma* and *Rhizoma Smilacis Glabrae* decoction could reduce UA in HUA rats, which is more efficient together than treated alone. The combination decoction may play its role in reducing UA by inhibiting activity of XOD, improving kidney damage and downregulating the expression of URAT1 in kidney.

## Figures and Tables

**Table 1:** Treatment for two stages.

Group	First stage		Group	Second stage	
	Gavage at 9 : 00 am (g/kg·bw)	Gavage at 2 : 00 pm (g/kg·bw)		Gavage at 9 : 00 am (g/kg·bw)	Gavage at 2 : 00 pm (g/kg·bw)
C	1% CMC-Na	Pure water	C	1% CMC-Na	Pure water
M	OXO 1.50 + ADE 0.05	Pure water	M	OXO 1.50 + ADE 0.05	Pure water
A	OXO 1.50 + ADE 0.05	Allopurinol 0.027	A	OXO 1.50 + ADE 0.05	Allopurinol 0.027
AR-L	OXO 1.50 + ADE 0.05	AR 0.50	PF-1	OXO 1.50 + ADE 0.05	AR 1.00 + RSG 1.00
AR-H	OXO 1.50 + ADE 0.05	AR 2.00	PF-2	OXO 1.50 + ADE 0.05	AR 2.00 + RSG 1.00
RSG-L	OXO 1.50 + ADE 0.05	RSG 1.25	PF-3	OXO 1.50 + ADE 0.05	AR 1.00 + RSG 2.00
RSG-H	OXO 1.50 + ADE 0.05	RSG 5.00	PF-4	OXO 1.50 + ADE 0.05	AR 2.00 + RSG 2.00

C, control; M, model; A, allopurinol; AR-L, low-dose of AR; AR-H, high dose of AR; RSG-L, low-dose of RSG; RSG-H, high dose of RSG; PF-1, combination 1; PF-2, combination 2; PF-3, combination 3; PF-4, combination 4; OXO, oteracil potassium; ADE, adenine.

**Table 2:** Body weights and liver/kidney weights for rats treated for 45 days in the first stage (*n* = 10).

Group	Body weight (g)	Relative liver weight (%)	Relative kidney weight (%)
C	386.5 ± 22.1	2.28 ± 0.11	0.62 ± 0.05
M	326.6 ± 20.7^▲▲^	2.23 ± 0.11	0.74 ± 0.05^▲▲^
A	343.8 ± 36.0^▲▲^	2.38 ± 0.05	0.72 ± 0.07^▲▲^
AR-L	351.9 ± 32.1^▲^	2.33 ± 0.15	0.71 ± 0.07^▲▲^
AR-H	338.5 ± 19.8^▲▲^	2.31 ± 0.14	0.68 ± 0.06
RSG-L	329.8 ± 35.1^▲▲^	2.32 ± 0.15	0.77 ± 0.10^▲▲^
RSG-H	250.2 ± 28.5^▲▲^^*∗∗*^	2.82 ± 0.34^▲^^*∗*^	0.75 ± 0.09^▲▲^

C, control; M, model; A, allopurinol; AR-L, low dose of AR; AR-H, high dose of AR; RSG-L, low dose of RSG; RSG-H, high dose of RSG. ▲ and ▲▲ indicate values significantly different from those of the control group at the levels of *P* < 0.05 and *P* < 0.01, respectively. ^*∗*^ and ^*∗∗*^ indicate values significantly different from those of the model group at the levels of *P* < 0.05 and *P* < 0.01, respectively.

**Table 3:** Liver biological index in each group in the first stage (*n* = 10).

Group	ALT (U/L)	AST (U/L)	T-BIL (*μ*mol/L)
C	49.33 ± 2.44	307.80 ± 49.36	1.00 ± 0.49
M	49.00 ± 6.54	275.00 ± 36.40	0.91 ± 0.30
A	54.62 ± 11.16	253.88 ± 39.15	1.33 ± 0.32
AR-L	50.83 ± 12.30	295.77 ± 54.13	0.87 ± 0.76
AR-H	51.44 ± 10.28	284.10 ± 42.75	1.27 ± 1.14
RSG-L	52.70 ± 10.13	301.45 ± 62.84	1.10 ± 0.52
RSG-H	93.67 ± 13.94^*∗∗*^	362.11 ± 46.89^*∗∗*^	3.50 ± 1.06^*∗∗*^

C, control; M, model; A, allopurinol; AR-L, low dose of AR; AR-H, high dose of AR; RSG-L, low dose of RSG; RSG-H, high dose of RSG; ALT, alanine aminotransferase; AST, aspartate aminotransferase; T-BIL, total bilirubin. ^*∗∗*^Significantly different from those of the model group at the level of *P* < 0.01.

**Table 4:** Percentage of decrease in UA of rats in the first stage (*n* = 10).

Group	Percentage of decrease in UA (%)		
	Day 15	Day 30	Day 45
C	—	—	—
M			
A	80.11	82.93	79.01
AR-L	−6.37	5.24	17.65
AR-H	4.22	9.08	19.87
RSG-L	7.04	7.53	18.74
RSG-H	20.46	25.73	34.92

C, control; M, model; A, allopurinol; AR-L, low dose of AR; AR-H, high dose of AR; RSG-L, low dose of RSG; RSG-H, high dose of RSG.

**Table 5:** Serum Cr and Ur levels in rats during 45 days in the first stage (*n* = 10).

Group	Cr (*μ*mol/L)				Ur (mmol/L)			
	Day 0	Day 15	Day 30	Day 45	Day 0	Day 15	Day 30	Day 45
C	39.52 ± 2.88	40.00 ± 4.13	42.07 ± 2.59	45.78 ± 4.85	4.90 ± 0.73	4.65 ± 0.70	5.03 ± 0.64	4.76 ± 0.82
M	39.41 ± 5.14	56.00 ± 2.59^▲▲^	59.47 ± 3.82^▲▲^	62.76 ± 2.93^▲▲^	4.87 ± 0.84	6.50 ± 0.86^▲▲^	6.71 ± 0.93^▲▲^	7.63 ± 0.49^▲▲^
A	39.39 ± 4.32	52.55 ± 3.33	59.00 ± 4.48	61.40 ± 4.36	4.95 ± 0.82	5.39 ± 0.66^*∗∗*^	5.70 ± 0.60^*∗*^	6.01 ± 0.76^*∗*^
AR-L	39.88 ± 4.87	57.01 ± 7.38	59.93 ± 5.07	64.55 ± 7.56	4.70 ± 0.79	6.62 ± 0.76	6.43 ± 0.72	7.12 ± 1.13
AR-H	38.56 ± 3.82	56.79 ± 5.03	59.19 ± 4.24	64.91 ± 5.88	4.78 ± 0.60	6.45 ± 0.93	6.66 ± 1.34	7.34 ± 2.20
RSG-L	37.70 ± 2.56	56.21 ± 5.04	58.91 ± 5.01	63.47 ± 7.61	4.68 ± 0.58	6.47 ± 0.88	6.05 ± 1.24	7.35 ± 1.49
RSG-H	38.89 ± 3.31	55.11 ± 7.42	53.92 ± 3.08^*∗∗*^	52.43 ± 9.65^*∗∗*^	4.74 ± 0.62	6.52 ± 0.69	5.76 ± 0.84^*∗*^	6.13 ± 1.27^*∗*^

C, control; M, model; A, allopurinol; AR-L, low dose of AR; AR-H, high dose of AR; RSG-L, low dose of RSG; RSG-H, high dose of RSG. ^▲▲^Significantly different from those of the control group at the level of *P* < 0.01. ^*∗*^ and ^*∗∗*^ indicate values significantly different from those of the model group at the levels of *P* < 0.05 and *P* < 0.01, respectively.

**Table 6:** Body weights and liver/kidney weights for rats treated for 45 days in the second stage (*n* = 10).

Group	Body weight (g)	Relative liver weight (%)	Relative kidney weight (%)
C	359.54 ± 6.39	2.56 ± 0.14	0.70 ± 0.06
M	359.95 ± 23.31	2.53 ± 0.15	0.82 ± 0.06^▲▲^
A	343.28 ± 31.62	2.60 ± 0.20	0.81 ± 0.06^▲▲^
PF-1	355.77 ± 24.32	2.63 ± 0.19	0.85 ± 0.06^▲▲^
PF-2	340.24 ± 27.60	2.57 ± 0.15	0.84 ± 0.07^▲▲^
PF-3	341.50 ± 25.28	2.66 ± 0.20	0.80 ± 0.08^▲▲^
PF-4	342.93 ± 21.75	2.54 ± 0.13	0.80 ± 0.03^▲▲^

C, control; M, model; A, allopurinol; PF-1, combination 1; PF-2, combination 2; PF-3, combination 3; PF-4, combination 4. ^▲▲^Significantly different from those of the control group at the level of *P* < 0.01.

**Table 7:** Liver biological index in each group in the second stage (*n* = 10).

Group	ALT (U/L)	AST (U/L)	T-BIL (*μ*mol/L)
C	49.40 ± 6.33	329.85 ± 68.82	0.83 ± 0.27
M	50.72 ± 13.71	348.69 ± 93.96	0.80 ± 0.37
A	52.08 ± 13.75	318.84 ± 50.50	0.88 ± 0.47
PF-1	56.82 ± 11.90	329.80 ± 74.53	0.89 ± 0.25
PF-2	50.92 ± 14.62	353.00 ± 57.18	0.87 ± 0.23
PF-3	52.00 ± 12.35	326.63 ± 87.02	0.92 ± 0.29
PF-4	50.60 ± 15.78	317.38 ± 76.14	0.92 ± 0.47

C, control; M, model; A, allopurinol; PF-1, combination 1; PF-2, combination 2; PF-3, combination 3; PF-4, combination 4; ALT, alanine aminotransferase; AST, aspartate aminotransferase; T-BIL, total bilirubin.

**Table 8:** Percentage of decrease in UA of rats in the second stage (*n* = 10).

Group	Percentage of decrease in UA (%)		
	Day 15	Day 30	Day 45
C	—	—	—
M			
A	76.82	76.63	76.56
PF-1	3.07	9.77	18.03
PF-2	5.87	13.06	35.04
PF-3	6.76	16.17	37.43
PF-4	5.59	18.20	54.89

C, control; M, model; A, allopurinol; PF-1, combination 1; PF-2, combination 2; PF-3, combination 3; PF-4, combination 4.

**Table 9:** Serum Cr and Ur levels of rats with different treatment during 45 days in the second stage (*n* = 10).

Group	Cr (*μ*mol/L)				Ur (mmol/L)			
	Day 0	Day 15	Day 30	Day 45	Day 0	Day 15	Day 30	Day 45
C	34.78 ± 3.27	34.36 ± 2.43	37.46 ± 2.99	39.23 ± 7.40	4.17 ± 0.80	3.91 ± 0.74	4.32 ± 0.64	4.83 ± 0.88
M	35.57 ± 4.31	46.07 ± 1.51^▲▲^	54.67 ± 5.01^▲▲^	61.57 ± 9.69^▲▲^	4.31 ± 0.49	5.23 ± 0.60^▲▲^	7.14 ± 1.19^▲▲^	10.20 ± 1.55^▲▲^
A	34.01 ± 2.24	34.06 ± 1.54^*∗∗*^	41.76 ± 3.67^*∗∗*^	50.98 ± 11.22^*∗∗*^	4.13 ± 0.66	4.49 ± 0.71^*∗∗*^	5.68 ± 0.82^*∗∗*^	7.05 ± 1.23^*∗∗*^
PF-1	32.78 ± 3.20	44.57 ± 1.82	52.84 ± 3.18	55.42 ± 12.61	3.85 ± 0.62	4.59 ± 0.47^*∗*^	6.84 ± 0.57	8.34 ± 2.54^*∗∗*^
PF-2	34.46 ± 5.23	46.46 ± 4.34	52.01 ± 3.20^*∗*^	50.60 ± 10.16^*∗*^	4.31 ± 0.90	4.95 ± 1.03	6.54 ± 0.75^*∗*^	8.36 ± 1.17^*∗∗*^
PF-3	33.52 ± 3.64	44.93 ± 3.28	49.96 ± 2.43^*∗∗*^	48.94 ± 14.55^*∗∗*^	4.12 ± 0.72	4.67 ± 0.68^*∗*^	6.14 ± 0.78^*∗∗*^	8.20 ± 2.31^*∗∗*^
PF-4	32.66 ± 2.54	45.76 ± 2.09	51.33 ± 2.75^*∗*^	48.52 ± 12.91^*∗∗*^	3.87 ± 0.73	4.73 ± 0.73	6.18 ± 0.76^*∗∗*^	7.85 ± 1.44^*∗∗*^

C, control; M, model; A, allopurinol; PF-1, combination 1; PF-2, combination 2; PF-3, combination 3; PF-4, combination 4. ^▲▲^Significantly different from those of control group at the level of *P* < 0.01. ^*∗*^ and ^*∗∗*^ indicate values significantly different from those of model group at the levels of *P* < 0.05 and *P* < 0.01, respectively.

## Data Availability

The data used to support the findings of this study are available from the corresponding author upon request.
